# Supplementation of SDF1 during Pig Oocyte In Vitro Maturation Improves Subsequent Embryo Development

**DOI:** 10.3390/molecules27206830

**Published:** 2022-10-12

**Authors:** Huaxing Zhao, Yazheng Dong, Yuxing Zhang, Xiao Wu, Xianjun Zhang, Yalin Liang, Yanan Li, Fang Zeng, Junsong Shi, Rong Zhou, Linjun Hong, Gengyuan Cai, Zhenfang Wu, Zicong Li

**Affiliations:** 1 National Engineering Research Center for Breeding Swine Industry, South China Agricultural University, Guangzhou 510030, China; 2 Department of Animal Genetics, Breeding and Reproduction, College of Animal Science, Guangzhou 510030, China; 3 Guangdong Provincial Key Laboratory of Agro-Animal Genomics and Molecular Breeding, South China Agricultural University, Guangzhou 510030, China; 4 College of Marine Science, South China Agricultural University, Guangzhou 510030, China; 5 Guangdong Wens Breeding Swine Technology Co., Ltd., Yunfu 527400, China; 6 State Key Laboratory for Conservation and Utilization of Subtropical Agro-Bioresources, South China Agricultural University, Guangzhou 510030, China

**Keywords:** pig, oocyte quality, IVM, embryo development, SDF1

## Abstract

The quality of in vitro matured oocytes is inferior to that of in vivo matured oocytes, which translates to low developmental capacity of embryos derived from in vitro matured oocytes. The developmental potential of in vitro matured oocytes is usually impaired due to oxidative stress. Stromal cell-derived factor-l (SDF1) can reduce oxidative stress and inhibit apoptosis. The aim of this study was to investigate the effects of SDF1 supplementation during pig oocyte in vitro maturation (IVM) on subsequent embryo development, and to explore the acting mechanisms of SDF1 in pig oocytes. We found that the IVM medium containing 20 ng/mL SDF1 improved the maturation rate of pig oocytes, as well as the cleavage rate and blastocyst rate of embryos generated by somatic cell nuclear transfer, in vitro fertilization, and parthenogenesis. Supplementation of 20 ng/mL SDF1 during IVM decreased the ROS level, increased the mitochondrial membrane potential, and altered the expression of apoptosis-related genes in the pig oocytes. The porcine oocyte transcriptomic data showed that SDF1 addition during IVM altered the expression of genes enriched in the purine metabolism and TNF signaling pathways. SDF1 supplementation during pig oocyte IVM also upregulated the mRNA and protein levels of YY1 and TET1, two critical factors for oocyte development. In conclusion, supplementation of SDF1 during pig oocyte IVM reduces oxidative stress, changes expression of genes involved in regulating apoptosis and oocyte growth, and enhances the ability of in vitro matured pig oocytes to support subsequent embryo development. Our findings provide a theoretical basis and a new method for improving the developmental potential of pig in vitro matured oocytes.

## 1. Introduction

In vitro matured oocytes can be used to generate various embryos through in vitro fertilization (IVF), parthenogenetic activation (PA), cloning or somatic cell nuclear transfer (SCNT), and genetic modification. These embryos have great application potential in livestock reproduction and breeding [1], human-assisted reproduction [2], and biomedicine [3]. However, the developmental competence of in vitro matured oocytes-derived embryos, such as the SCNT [4,5] and IVF [6,7] embryos, is usually lower than those produced by the in vivo matured oocytes. The developmental ability of embryos mainly depends on the quality of oocytes. Thus, the lower developmental rate of in vitro matured oocytes-derived embryos indicates that the quality of in vitro matured oocytes is inferior to that of in vivo matured oocytes.

The in vivo matured oocytes develop in hypoxic follicles, and the follicular fluid contains free radical scavengers and antioxidants that protect the in vivo matured oocytes against oxidative damage [8,9]. On the other hand, in vitro maturation (IVM) of oocytes occurs in a hyperoxic environment that lacks free radical scavengers and antioxidants [10], thereby exposing the oocytes to severe oxidative stress [11]. Hypoxic culturing conditions can improve the quality of in vitro matured pig oocytes [12,13]. In addition, some studies have shown that the level of oxidative stress is lower in the in vivo matured oocytes compared to the in vitro matured oocytes [14,15], which corresponds to higher levels of the antioxidant glutathione in the former [16]. In addition, the in vivo matured oocytes have healthier mitochondria, along with lower apoptosis rates and expression levels of pro-apoptotic genes, compared to the in vitro matured oocytes [17]. This is consistent with the fact that the vicious cycle between oxidative stress and mitochondrial dysfunction eventually leads to oocyte apoptosis in vitro [18,19]. Therefore, oxidative stress is a significant factor that impairs the developmental potential of in vitro matured oocytes.

Stromal cell-derived factor-l (SDF1), also called C-X-C motif chemokine ligand 12 (CXCL12), is an antioxidant and free radical scavenger [20,21,22]. It can suppress apoptosis of cultured cells by reducing oxidative stress [20,23]. Human leukemia cells cultured in the presence of SDF1 produce low levels of ROS due to lower mitochondrial metabolism [24]. The level of SDF1 in human [25] and porcine follicular fluid [22] is predictive of the outcome of embryonic development. Previous studies have shown that supplementation of SDF1 alone during ovine oocyte IVM can enhance subsequent PA embryo development, and combined supplementation of SDF1 with other factors to porcine oocyte IVM medium can improve subsequent embryo development [26,27].

Although SDF1 can reduce oxidative stress in cultured cells, and is related to follicle and embryo development, it is unclear whether supplementation of SDF1 alone during pig oocyte IVM can improve the quality or developmental potential of treated oocytes. The aim of this study was to evaluate the effects of SDF1 supplementation during porcine oocyte IVM on subsequent embryo development.

## 2. Results

### 2.1. SDF1 Concentration Was Increased in IVM Medium and Follicular Fluid during Pig Oocyte Maturation

**Figure 1:**
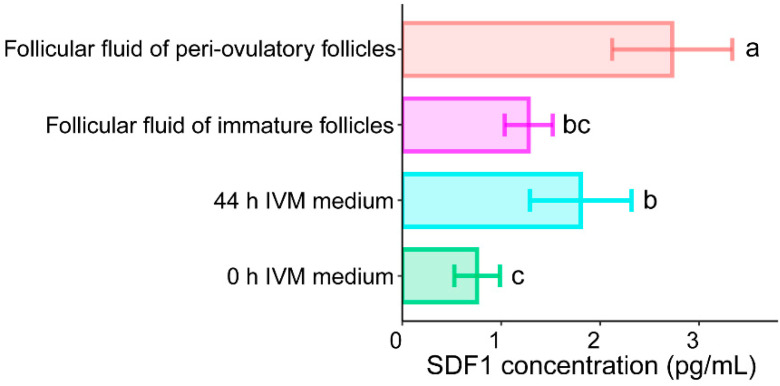
The SDF1 concentration in porcine oocyte IVM medium and follicular fluid. Values are presented as mean ± SD. Values labeled with different letters differ at *p* < 0.05.

To investigate the changes of SDF1 concentration in IVM medium and follicular fluid during in vitro or in vivo pig oocyte maturation, we analyzed SDF1 concentration in 0 h IVM medium, 44 h IVM medium, follicular fluid of immature follicles, and follicular fluid of peri-ovulatory follicles. The results showed that SDF1 level was higher in 44 h IVM medium than in 0 h IVM medium, and was higher in follicular fluid of peri-ovulatory follicles than in follicular fluid of immature follicles (Figure 1). This suggests that SDF1 was expressed and secreted from porcine COCs during both in vitro and in vivo maturation. However, the SDF1 concentration in follicular fluid of peri-ovulatory follicles was significantly higher than that in 44 h IVM medium (Figure 1), suggesting that in vitro matured pig COCs produced less SDF1 than in vivo matured counterparts.

### 2.2. SDF1 Supplementation during Porcine Oocyte IVM Improved Subsequent Embryo Development

**Figure 2:**
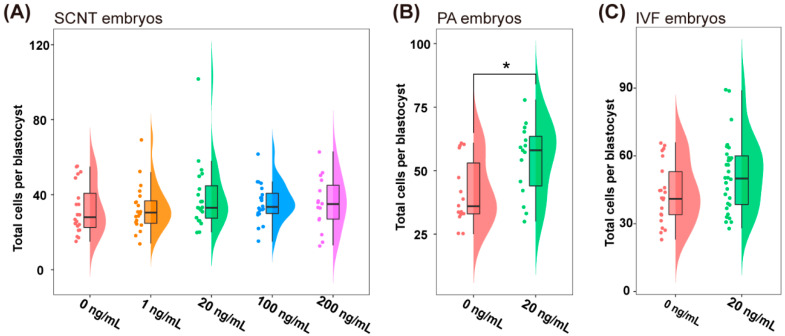
Effects of supplementation of SDF1 during pig oocyte IVM on the total cell number per blastocyst of SCNT (**A**), PA (**B**), and IVF (**C**) embryos. “*” represents *p* < 0.05.

To investigate the effects of SDF1 supplementation during IVM on the maturation and quality of pig oocytes, porcine COCs were cultured in IVM medium supplemented with different concentrations of recombinant SDF1 (0, 1, 20, 100, and 200 ng/mL). As shown in Table 1, 20 ng/mL SDF1 significantly increased the maturation rate of the oocytes. Although the difference in oocyte maturation rate between the 200 ng/mL SDF1 supplementation and control groups was not statistically significant, 200 ng/mL SDF1 treatment tended to decrease the pig oocyte maturation rate (Table 1). The matured oocytes were subsequently used for SCNT to ascertain the effect of SDF-1 supplementation on embryo development. The addition of 20 ng/mL and 100 ng/mL SDF1 during IVM significantly improved the cleavage rate of cloned embryos (Table 2), and 20 ng/mL SDF1 also considerably increased the blastocyst rate of cloned pig embryos (Table 2). Similarly, the addition of 20 ng/mL of SDF1 during in vitro oocyte maturation improved the blastocyst formation rates of the PA and IVF embryos (Table 3 and Table 4). Supplementation of 20 ng/mL SDF1 during pig oocyte IVM increased the blastocyst cell counts of PA embryos and tended to improve the blastocyst cell number of SCNT and IVF embryos (Figure 2).

### 2.3. SDF1 Supplementation during IVM Decreased Oxidative Stress in Pig Oocytes

**Figure 3:**
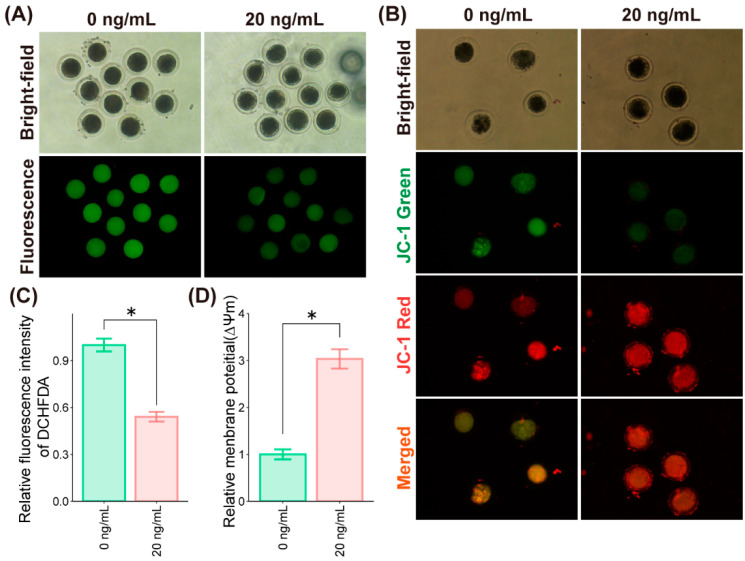
SDF1 supplementation alleviates oxidative stress in the porcine oocytes during IVM. (**A**) Representative images of the control and SDF1-treated oocytes incubated with H_2_DCFDA showing ROS production. (**B**) Representative images of the control and SDF1-treated oocytes incubated with JC-1 for ∆Ψm measurements. (**C**) ROS levels in the indicated groups were calculated in terms of the intensity of green fluorescence. (**D**) ∆Ψm in the indicated groups was calculated as the ratio of red/green fluorescence intensity. Values are presented as mean ± SD. “*” represents *p* < 0.05.

To further explore the mechanisms underlying the beneficial effects of SDF1 on porcine oocyte maturation and the generation of embryos, we analyzed ROS levels during IVM. Addition of 20 ng/mL of SDF1 during IVM significantly reduced ROS levels in the porcine oocytes compared to that in the untreated controls (Figure 3A,C), indicating that SDF1 alleviates oxidative stress in porcine oocytes. High levels of ROS trigger mitochondrial dysfunction [28,29], which can impair the metabolism of oocytes [30,31]. Therefore, we also measured the mitochondrial membrane potential (ΔΨm), an indicator of mitochondrial activity, at 24 h intervals during oocyte maturation. The ΔΨm of the untreated oocytes was lower than that of the 20 ng/mL SDF1-treated oocytes (Figure 3B,D). These results indicate that supplementing IVM medium with SDF1 can significantly improve mitochondrial function in pig oocytes.

**Figure 4:**
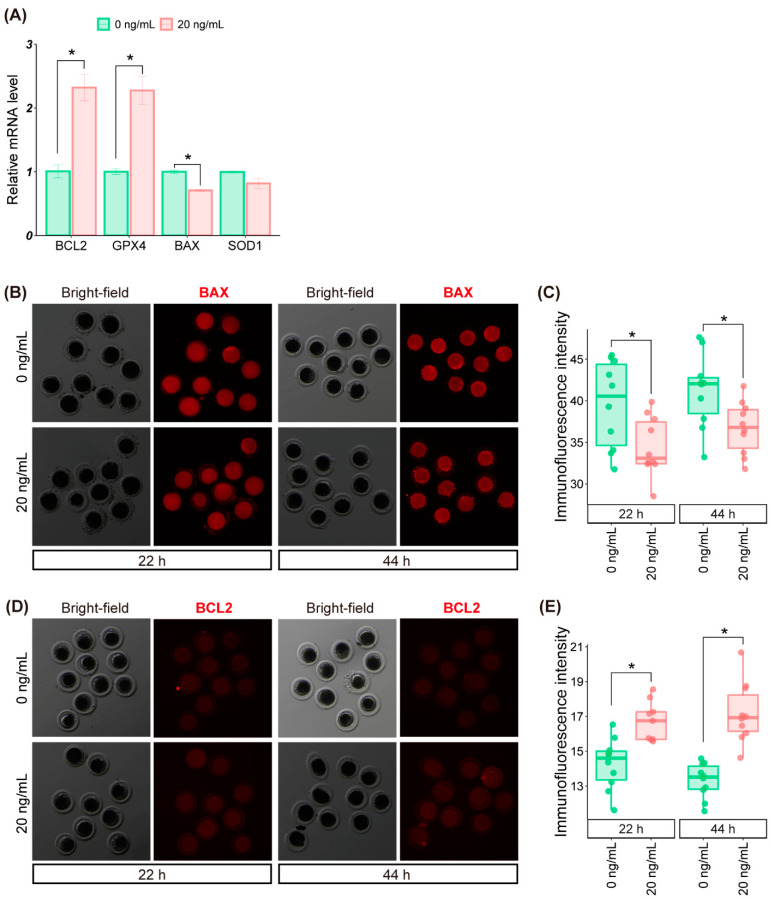
Expression of apoptosis and oxidative stress-related genes in control and SDF1-treated pig oocytes. (**A**) Relative mRNA expression levels of apoptosis and oxidative stress-related genes in porcine oocytes. BAX (**B**) and BCL2 (**D**) protein levels in pig oocytes. Quantification of BAX (**C**) and BCL2 (**E**) proteins in pig oocytes. “*” represents *p* < 0.05.

We next analyzed the expression levels of genes involved in apoptosis and oxidative stress, including BCL2-associated X (*BAX*) [32], superoxide dismutase 1 (*SOD1*) [33], B-cell lymphoma 2 (*BCL2*) [34], and glutathione peroxidase 4 (*GPX4*) [35] in porcine oocytes. As shown in Figure 4A, the pro-apoptotic *BAX* was downregulated, whereas the anti-apoptotic *BCL2* was upregulated in the SDF1-treated oocytes compared to the untreated controls. Furthermore, while SDF1 supplementation increased the mRNA levels of the antioxidant gene *GPX4*, it had no significant effect on the expression of *SOD1* gene (Figure 4A). We further compared the protein levels of BAX and BCL2 in oocytes between the untreated and SDF1-treated groups. The BAX protein level was decreased (Figure 4B,C) and the BCL2 protein abundance was increased (Figure 4D,E) in SDF1-treated oocytes during IVM, indicating that the changes in mRNA level was consistent with the alterations in protein level of BAX and BCL2.

### 2.4. SDF1 Supplementation during IVM Alters the Transcriptome of Porcine Oocytes

**Figure 5:**
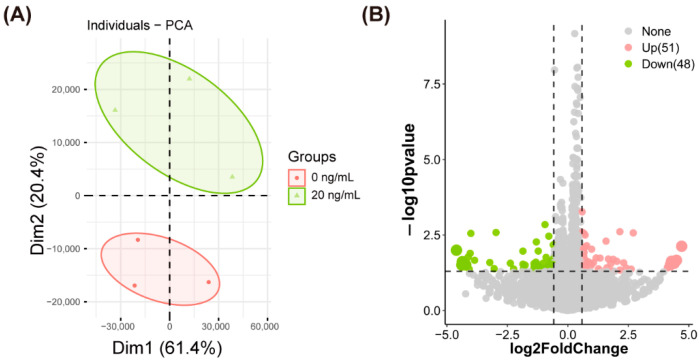
(**A**) Principal component analysis (PCA) of sequenced pig matured oocytes in vitro samples from control and 20 ng/mL SDF1-treated groups and (**B**) volcano plot of differentially expressed genes between two groups.

**Figure 6:**
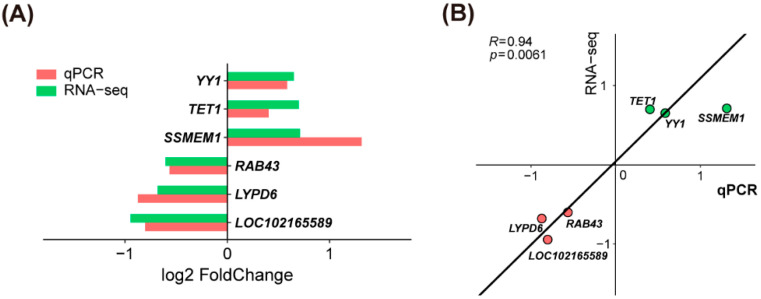
Verification of randomly selected RNA-seq-identified DEGs by qPCR.

To determine if SDF1 regulates global gene expression in porcine in vitro matured oocytes, we analyzed the transcriptomes of the untreated and SDF1-treated oocytes by RNA sequencing. Approximately 322.6 million raw reads and 310.86 million clean reads were obtained from six control and 20 ng/mL SDF1-treated samples (Table 5). The rate of Q30 clean reads bases ranged from 93.36–93.83% (Table 5). Of the total reads, 65.24–82.57% were mapped to the reference genome, and the percentages of uniquely mapped reads ranged from 63.7–80.6% (Table 5). The principal component analysis (PCA) showed that different samples in the same group were clustered, suggesting fine separation between the two groups, and sample reproducibility within the same group (Figure 5A). Differentially expressed genes (DEGs) were screened based on the *p*-value < 0.05 and fold change > 1.5. A total of 99 DEGs (51 up-regulated and 48 down-regulated) were identified in the SDF1-treated group relative to the control (Table S2 and Figure 5B). Six DEGs, including YY1, TET1, and four other randomly selected DEGs, were validated by qPCR, and their expression levels detected by qPCR and RNA-seq were positively correlated (Figure 6), indicating that the RNA-seq data was reliable.

### 2.5. Go and KEGG Analysis of DEGs Identified by RNA-Seq

**Figure 7:**
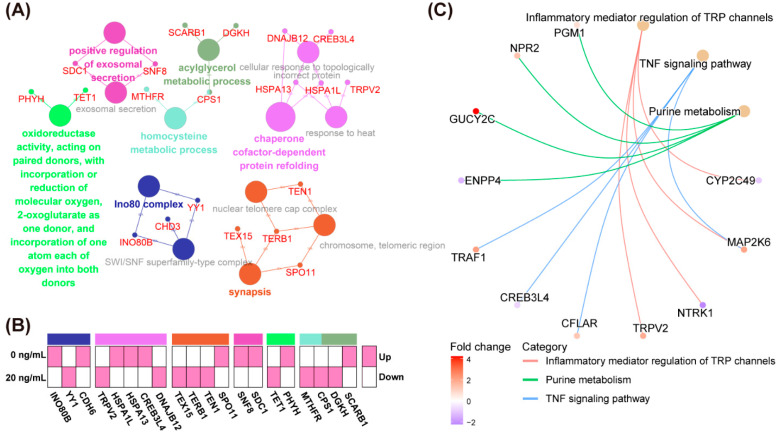
Functional enrichment analysis of DEGs between control and 20 ng/mL SDF1-treated groups. (**A**) The GO term enrichment analysis of DEGs. Different colors of nodes refer to the functional annotation of ontologies, the gray fonts represent non-significant terms, and the red fonts represent DEGs in significantly enriched GO terms. (**B**) The up-regulated and down-regulated DEGs in significantly enriched GO terms. (**C**) The KEGG term enrichment analysis of DEGs.

To further explore the mechanisms of SDF1 in regulating pig oocyte maturation in vitro, we functionally annotated the 99 DEGs using the Cytoscape software with ClueGO plug-in. Twenty DEGs were significantly enriched in seven pathways, including the chaperone cofactor-dependent protein refolding, synapsis, acylglycerol metabolic process, oxidoreductase activity, homocysteine metabolic process, positive regulation of exosomal secretion, and Ino80 complex pathways (Figure 7A). Figure 7B demonstrates the up-regulated and down-regulated 20 DEGs in seven significantly enriched pathways. Moreover, KEGG analysis indicated that the DEGs were significantly enriched in three signaling pathways, including inflammatory mediator regulation of TRP channels, TNF signaling pathway, and purine metabolism (Figure 7C).

### 2.6. SDF1 Supplementation during IVM Upregulated the Levels of TET1 and YY1 Proteins in Pig Oocytes

**Figure 8:**
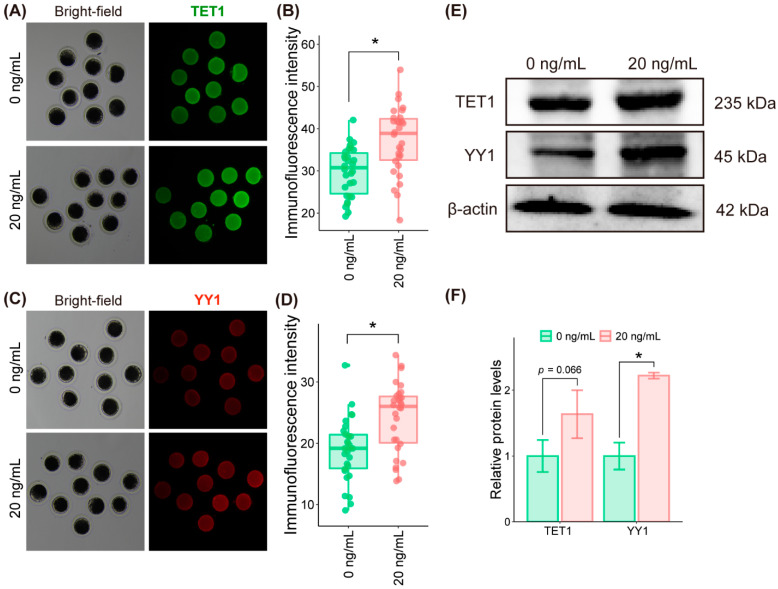
Comparison of TET1 and YY1 protein levels between the 20 ng/mL SDF1-treated and control porcine oocyte. Detection of TET1 (**A**) and YY1 (**C**) proteins in matured oocytes by immunofluorescence staining. Quantification of TET1 (**B**) and YY1 (**D**) proteins measured by immunofluorescence staining. (**E**) Detection of TET1 and YY1 proteins in matured oocytes by western blotting. (**F**) Quantification of TET1 and YY1 proteins measured by western blotting. “*” represents *p* < 0.05.

The effect of SDF1 supplementation on the protein level of the DEGs associated with oocyte development was also analyzed. We compared the expression levels of two DEG-encoded proteins, TET1 and YY1, between the untreated and the SDF1-treated groups by immunofluorescence staining and western blotting. Consistent with the corresponding mRNA levels, both TET1 and YY1 proteins were upregulated in the SDF1-treated oocytes compared to the control oocytes (Figure 8). These results suggested that SDF1 treatment during IVM alters the expression of several genes at the mRNA as well as the protein level.

## 3. Discussion

We found in this study that pig COCs expressed SDF1 and secreted it into IVM medium or follicular fluid during in vitro or in vivo maturation. This implies that SDF1 is needed for pig oocyte maturation. Nevertheless, the SDF1 level in 44 h IVM medium was lower than that in follicular fluid of peri-ovulatory follicles, suggesting that the amount of SDF1 produced by in vitro matured pig oocytes is insufficient compared with that synthesized by in vivo matured pig oocytes.

A study demonstrated that the ovine oocyte maturation rate is improved by supplementation of IVM medium with 3 and 10 ng/mL but not with 1, 30, and 300 ng/mL of SDF1, and supplementation of 10 ng/mL SDF1 during ovine oocyte IVM increased the subsequent development of PA embryos, including the 2-cell, 4-cell, 8-cell, morula, and blastocyst rates [27]. Supplementation of 10 ng/mL SDF1 during IVM improved the maturation rate of pig denuded oocytes and tended to increase the maturation rate of pig oocytes with cumulus cells, yet 1, 5, 25, and 100 ng/mL SDF1 treatment had no influence on the in vitro maturation rate of both types of pig oocytes [26]. In this study, we found that 20 ng/mL but not 1, 100, and 200 ng/mL of SDF1 treatment enhanced pig oocyte maturation in vitro, and 20 ng/mL SDF1 supplementation during IVM increased the ability of pig oocyte to form embryos via SCNT, PA, and IVF. The effective SDF1 concentration, and the positive influence of SDF1 on in vitro oocyte maturation and subsequent embryo development, are similar in the above-mentioned two reports and the present study. The three studies suggest that the optimal SDF1 concentration for supplementation to oocyte IVM medium is 3 to 20 ng/mL, since no positive effect on oocyte maturation and developmental potential was observed when the final concentration of added SDF1 is outside the range of 3 to 20 ng/mL.

We showed that the effective SDF1 concentration for adding to the pig oocyte IVM medium was 20 ng/mL. This concentration was much higher than that found in the follicular fluid of in vivo mature follicles of sows, which was about 3 pg/mL. The difference in the concentration of supplied SDF1 in the IVM medium and the level of endogenous SDF1 in mature follicles could be explained by the following reasons: (1) A mature follicle of a sow contains about 6 pg of endogenous SDF1 protein since the follicular fluid volume of a mature follicle of a sow is about 2 mL. However, during in vitro maturation, endogenous SDF1 could be continuously secreted by the follicular cells or supplied by the blood circulation, and continuously consumed by the oocytes. This means that the consumption amount of endogenous SDF1 for an oocyte within a follicle during the whole in vivo maturation process could be much higher than the amount of 6 pg, which was measured after the follicles reach maturation. (2) The pig oocyte IVM experiment in this study was performed by culturing 50 oocytes on one well of a four-well plate with 0.5 mL of IVM medium containing 20 ng/mL of recombinant SDF1. This means that each pig oocyte consumes 0.2 ng or 200 pg of recombinant SDF1 during the whole in vitro maturation process. However, the SDF1 protein used in this study was produced by bacteria, and its bioactivity may be lower than that of the endogenous SDF1 protein. When the bacteria-produced SDF1 protein was used for in vitro pig oocyte maturation, a higher amount than the endogenous amount of SDF1 should be added to the IVM medium. (3) The environment of in vivo oocyte maturation is very different from that of in vitro oocyte maturation, which may lead to two types of oocytes that require different amounts of SDF1 protein for maturation, as observed in this study.

Our results indicated that SDF1 supplementation reduced ROS levels in the porcine oocytes during IVM. Consistent with the fact that ROS in pig oocytes are mainly produced in the mitochondria and trigger damage to the latter [19,36], SDF1-mediated reduction in ROS levels of pig oocytes enhanced the mitochondrial activity of oocytes by increasing the mitochondrial membrane potential. High ROS levels also activate the apoptosis pathways in porcine oocytes [37,38,39]. In the present study, we found that SDF1 downregulated the mRNA level of the pro-apoptotic gene *BAX*, and upregulated the mRNA level of the anti-apoptotic genes *BCL2* and *GPX4* in the treated pig oocytes. In addition, the BAX protein level was downregulated and the BCL2 protein abundance was upregulated in SDF1-treated group oocytes compared with that in control oocytes. The changes in the transcript and protein levels of apoptosis-related genes might contribute to the enhancement in developmental potential of SDF1-treated pig oocytes.

RNA-seq analysis of the untreated and SDF1-treated oocytes further revealed that the expression levels of several stress-related genes, including *CYR61*, *HSPA1L*, and *TEX15*, were altered after SDF1 supplementation. The abundance of *CYR61*, an antioxidant gene acting through integrin-mediated induction [40], was lower in the oocytes treated with SDF1. The stress-responsive gene *HSPA1L* was also down-regulated in the SDF1-treated oocytes [41]. *TEX15*, a DNA damage repair gene activated in response to oxidative stress [42,43], was upregulated in the SDF1-treated versus the control oocytes.

The KEGG enrichment analysis showed that the DEGs between the control and SDF1-treated oocytes were enriched in three signaling pathways: inflammatory mediator regulation of TRP channels, TNF signaling pathway, and purine metabolism. TNF signaling mediates pig cell apoptosis [44,45], which is suppressed by CFLAR, a central regulator of the TNF signaling pathway [44,46]. *CFLAR* expression was up-regulated in the SDF1-treated group, suggesting that SDF1 protects the oocytes against apoptosis by downregulating the TNF signaling pathway. In addition, the beneficial effects of SDF1 on pig in vitro matured oocytes and the subsequent development of embryos may be related to the changes in purine metabolism, which affects oocyte quality and developmental potential in sows [47].

Only a small number of genes in the genome are transcriptionally active, but most of the genes in the genome are transcriptionally silent and their mRNAs are degraded during the oocyte maturation process [48,49,50,51]. The SDF1 supplementation-induced changes in the mRNA abundance of DEGs in matured pig oocytes might be mainly regulated by post-transcriptional modification, such as polyadenylation, rather than transcriptional activation or inhibition. SDF1 treatment increased the mRNA and protein levels of YY1 and TET1, two factors that are critical to oocyte quality. YY1 is required for oocyte maturation, granulosa cell expansion, and oocyte-granulosa cell communication [52], whereas TET1 participates in female germ cell growth and embryo genome reprogramming [53]. TET1 deficiency leads to defective DNA demethylation and reduces expression of a subset of meiotic genes in oocytes [54]. TET1 plays a role in regulating the genome activation and pluripotency of pig early embryos [55,56]. The upregulation of the mRNA and protein levels of YY1 and TET1 in SDF1-treated pig oocytes might enhance the ability of the matured oocytes to support subsequent embryo development.

The early developmental capacity of in vitro produced embryos is mainly determined by the quality of oocytes used to generate the embryos [26,57]. Theoretically, both the cytoplasmic and nuclear factors in the matured oocytes can impact the development of IVF and PA embryos. However, only the factors in the cytoplasm of enucleated matured oocytes have influence on subsequent SCNT embryo reprogramming. In this study, SDF1 supplementation during oocyte IVM not only improved IVF and PA embryo development, but also enhanced SCNT embryo formation. This finding suggests that some molecules in the cytoplasm rather than in the nucleus of in vitro matured oocytes are responsible for the enhancement in embryo development induced by SDF1 supplementation during oocyte IVM. YY1 and TET1 are possibly two of the key oocyte cytoplasmic molecules that contributed to SDF1 addition during oocyte IVM-induced subsequent embryo development, because they play important roles in oocyte quality and their protein levels in the cytoplasm of SDF1-treated oocytes are increased, as shown in this study.

## 4. Materials and Methods

### 4.1. Chemicals

Unless otherwise mentioned, all chemicals used in the experiments were procured from Sigma-Aldrich Company (St. Louis, MO, USA).

### 4.2. Preparation of IVM Medium

The IVM medium was reported by Jeong et al. [58], but we made some adjustments. Briefly, TCM-199 medium (Sigma, St. Louis, MO, USA) was supplemented with 10% (*v/v*) fetal bovine serum (FBS; Gibco, Grand Isle, NY, USA), 10% (*v/v*) porcine follicular fluid collected from antral follicles (diameter ≥ 8 mm) of slaughter house-derived ovaries, 0.1% polyvinyl alcohol, 3.05 mM d-glucose, 0.91 mM sodium pyruvate, 10 ng/mL epidermal growth factor, 0.6 mM cysteine, 10 IU/mL pregnant mare serum gonadotropin, and 10 IU/mL human chorionic gonadotropin. The *E. coli*-expressed recombinant SDF1 (Cat #: C121, Novoprotein, Shanghai, China) was dissolved in DPBS containing 0.1% BSA. Then the concentration was adjusted and added into the IVM medium to make the final concentrations of 0 ng/mL, 1 ng/mL, 20 ng/mL, 100 ng/mL, and 200 ng/mL, respectively.

### 4.3. Preparation of In Vitro Matured Oocytes

Ovaries of gilts were obtained from a local slaughterhouse and transported to the laboratory in 0.9% saline (*w/v*) within three hours. The NaCl solution was supplemented with penicillin-G (100 IU/mL) and streptomycin sulfate (100 mg/L) at 30 °C–35 °C. Follicular fluid with the oocytes was aspirated from the antral follicles (3–6 mm in diameter) and collected into a 50 mL centrifuge tube using an 18-gauge needle connected to a 10 mL disposable syringe. Cumulus-oocyte complexes (COCs) were recovered under a stereomicroscope. The samples with at least three layers of compact cumulus cells and a homogenous cytoplasm were selected for IVM. The selected COCs were washed thrice with HEPES-buffered Tyrode’s medium containing 0.05% (*w/v*) polyvinyl alcohol. Approximately 50–60 oocytes were transferred to each well of a four-well Nunc dish into 500 μL IVM media supplemented with different concentrations of SDF1, and cultured at 38.5 °C with 5% CO_2_ for 44 h in a humidified atmosphere. The COCs were treated with DPBS containing 1 mg/mL hyaluronidase to remove the surrounding cumulus cells. The oocytes were observed under a stereoscope, and those with the first polar body in the perivitelline space were considered as mature. The mature oocytes with intact cell membranes and clear perivitelline space were selected for SCNT, IVF, and PA.

### 4.4. Enzyme-Linked Immune Sorbent Assay (ELISA)

Fresh porcine COCs IVM medium (0 h IVM medium) was collected into frozen storage tubes, which were immediately stored in liquid nitrogen. The IVM medium of porcine COCs cultured for 44 h (44 h IVM medium) was collected into centrifuge tubes, centrifuged at 3000 rpm for 5 min, and the supernatant was immediately transferred into frozen tubes for storage in liquid nitrogen. The collection of follicular fluid was performed as previously described [5]. The follicular fluid of peri-ovulary follicles was collected from sow ovaries (the point of ovulation was observed) and follicular fluid of immature follicles (3 mm < diameter < 8 mm) was collected from gilt ovaries. The porcine SDF1 ELISA assays were performed following the manufacturer’s instructions (Jinmei, Jiangsu, China), and detected using a microplate reader (BioTek, Burlington, VT, USA).

### 4.5. Preparation of Donor Cells

Fibroblast cells were obtained from a Duroc boar by ear biopsy. After a quick rinse in 75% ethanol and DPBS supplemented with penicillin (100 IU/mL) and streptomycin (100 μg/mL), the ear tissues were minced into 1 to 2 mm pieces in DMEM supplemented with 10% FBS. Fragments of ear tissue were seeded into 100 mm cell culture dishes in 6 mL FBS-supplemented DMEM (Invitrogen, Grand Island, NY, USA), which were then placed in a humidified incubator at 38.5 °C with 5% CO_2_. The culture medium was changed every other day. The fibroblasts were harvested with 0.25% trypsin-EDTA (Invitrogen, Grand Island, NY, USA) and passaged at 1:3 split ratios (passage number 1, P1). The fibroblast cells from passages 3–5 were frozen in liquid nitrogen (freezing medium—50% FBS, 40% DMEM, and 10% DMSO). Before SCNT, the cells were thawed and cultured as described above in FBS-supplemented DMEM for 3–4 days until they reached 80–90% confluence. Adherent cells from passages 6–10 were harvested with 0.05% trypsin for 1 min and used for SCNT.

### 4.6. Production of Embryos

The production of somatic cell nuclear transfer (SCNT) embryos was performed as previously described [59], but with some adjustments. The matured oocytes and a small number of donor cells were suspended in T2 medium (TCM-199 plus 2% FBS) containing 7.5 μg/mL cytochalasin B (CB). The oocytes were enucleated by microinjection using a 17 μm diameter needle (Lingen Precision Medical Products Co., Ltd., Shanghai, China). The microinjection needle was inserted into the oocyte to aspirate the first polar body along with approximately 15% of the adjacent cytoplasm that contains the genomic material. Enucleated oocytes were stained with 1 g/mL Hoechst 33,342 and examined under UV light irradiation. Non-enucleated or incompletely enucleated oocytes with blue staining signal inside the cytoplasm were immediately discarded. Only those completely enucleated oocytes were used for subsequent SCNT. Donor cells with a round and slightly burr-like shape were aspirated into the injection needle, and one donor cell was injected into the perivitelline space of each enucleated oocyte. The reconstructed oocytes were washed three times in PZM-3 [60] and then electrically fused using two direct current pulses of 150 V/mm for 50 μs in 0.28 mol/L mannitol supplemented with 0.1 mM MgSO_4_ and 0.01% PVA. The fused oocytes were cultured in PZM-3 medium for 1 h before electro-activation. The reconstructed oocytes were activated by two direct current pulses of 100 V/mm for 20 μs in 0.28 mol/L mannitol supplemented with 0.1 mM MgSO_4_ and 0.05 mM CaCl_2_. The activated embryos were incubated in PZM-3 medium supplemented with 5 mg/mL CB for 4 h and then transferred into fresh PZM-3 medium at 38.5 °C under 5% CO_2_ and saturated humidity.

The production of parthenogenetic activation (PA) embryos followed a reported study [61], but some parameters were adjusted. The matured oocytes were washed three times in PZM-3 and then electrically activated using two direct current pulses of 150 V/mm for 50 μs in 0.28 mol/L mannitol supplemented with 0.1 mM MgSO_4_ and 0.01% PVA. The activated oocytes were then cultured in PZM-3 medium supplemented with 5 mg/mL CB for 4 h at 38.5 °C under 5% CO_2_ and saturated humidity.

The procedure for production of in vitro fertilization (IVF) embryos was previously described [62]. The matured oocytes were transferred to mTBM medium (11.0 mM glucose, 5.0 mM sodium pyruvate, 113.1 mM NaCl, 7.5 mM CaCl_2_·2H_2_O, 3.0 mM KCl, and 20.0 mM Tris) supplemented with 2 mg/mL BSA and 2.5 mM caffeine. The fresh semen (Guangdong Wens Breeding Swine Technology Co., Ltd., Yunfu, China) was washed three times by centrifugation with DPBS containing 0.1% BSA at 1500 rpm for 5 min. The spermatozoa were resuspended and capacitated with mTBM in the CO_2_ incubator for 30 min. The capacitated sperm were added to the well containing matured oocytes with a final concentration of 1 × 10^5^ sperm/mL for 6 h at 38.5 °C under 5% CO_2_ and saturated humidity.

The embryos generated by SCNT, PA, and IVF were transferred to fresh PZM-3 for extended culture, and the cleavage and blastocyst rates were assessed two and six days after embryo production. The total blastocyst cell count was determined by Hoechst 33,342 staining under an epifluorescence microscope (Ti2; Nikon).

### 4.7. RNA Sequencing and Bioinformatics Analysis

The matured oocytes were washed thrice with DPBS at 37.5 °C. Fifty randomly selected oocytes were pooled and transferred into a 200 μL PCR tube containing 10 μL RNA lysis buffer, and then stored at −80 °C. Oocyte RNA extraction, library construction, and transcriptome sequencing were conducted by OE Biotech Co., Ltd. (Shanghai, China). Each group had three biological replicates, and six samples were sequenced. Filtered clean reads were mapped to the Sus scrofa 11.1 reference genome. The differentially expressed genes (DEGs) between the control (0 ng/mL) and SDF1-supplemented (20 ng/mL) groups were identified using R package DEseq2 [63]. Gene Ontology (GO) analysis of the DEGs was performed by ClueGO and CluePedia plug-in of Cytoscape. The R package clusterProfiler [64] was used for Kyoto Encyclopedia of Genes and Genomes (KEGG) analysis.

### 4.8. Real-Time Quantitative Polymerase Chain Reaction (qPCR) Analysis

Total RNA was extracted from the matured oocytes using the complete RNA Kit (Omega, Atlanta, Georgia, USA), and quantified using NanoDrop 2000 Spectrophotometer (Thermo Fisher Scientific, Wilmington, DE, USA). Only intact RNA samples that did not show any signs of degradation were stored at −80 °C till subsequent analysis. The cDNA was synthesized from 1 μg total RNA using the PrimeScript RT Reagent Kit (TaKaRa, Otsu, Japan), and qPCR was performed using the PowerUp™ SYBR Green Mix (Applied Biosystems, MA, USA) according to the manufacturers’ protocols. The primer sequences of all transcripts are listed in Table S1. The expression of each target gene was quantified relative to that of the internal control gene (*RN18S*) using the equation R = 2 − [ΔCt sample − ΔCt control].

### 4.9. Measurement of ROS Levels in Oocytes

The ROS levels in the oocytes were measured using the ROS Assay Kit (Yesen, Shanghai, China) as per the manufacturer’s instructions. The oocytes were incubated with 10 µM 2′,7′-dichlorodihydrofluorescein diacetate (D_2_DCFDA) in M199 medium at 38.5 °C for 15 min in the dark. After washing twice with M199 medium at room temperature, the oocytes were transferred into 30 µL DPBS droplets, and the fluorescence was observed using an epifluorescence microscope (Ti2; Nikon). The fluorescence intensity was analyzed by ImageJ software (National Institutes of Health, Bethesda, MD, USA).

### 4.10. Detection of Mitochondrial Membrane Potential (ΔΨm) in Oocytes

The ΔΨm in the oocytes was measured using JC-1 mitochondrial membrane potential assay kit (Abcam, Cambridge, UK). The oocytes were incubated with 10 µM JC-1 in medium at 37 °C for 40 min in the dark. The samples were washed thrice with DPBS, and the fluorescence intensity was detected with an epifluorescence microscope using ImageJ software. The ΔΨm was calculated as the ratio of red to green fluorescence.

### 4.11. Immunofluorescent Staining of In Vitro Matured Oocytes

The matured oocytes were fixed with 4% paraformaldehyde (Beyotime, Shanghai, China) for 15 min and permeabilized in 0.5% Triton X100 (Beyotime, Shanghai, China) for 20 min. After blocking with QuickBlock™ blocking buffer (Beyotime, Shanghai, China) for 30 min, the oocytes were washed thrice with DPBS and incubated overnight with primary antibodies targeting BAX (Santa Cruz, Dallas, USA), BCL2 (Santa Cruz, Dallas, TX, USA), TET1 (Bioss, Beijing, China), and YY1 (Proteintech, Rosemont, Chicago, IL, USA) diluted 1:100 at 4 °C. The oocytes were washed thrice with DPBS, and then incubated with Alexa Fluor 488 goat anti-rabbit IgG or Cy3–conjugated affiniPure goat anti-mouse IgG diluted 1:200.

### 4.12. Western Blot

Protein concentration of the oocytes was determined using Micro BCA Protein Assay Kit (CWBIO, China). The proteins and color pre-stained protein marker were separated on sodium 12% SDS/PAGE gels (CWBIO, Beijing, China), and transferred into polyvinylidene difluride membranes (Merck Millipore, Darmstadt, Germany). The membrane was blocked by 5% skimmed milk powder (BD, Franklin Lake, NJ, USA) using a shaker, and incubated with antibodies at 4 °C. The proteins were performed using Eecl Western blot kit (CWBIO, Beijing, China), and band densities were analyzed with imageJ software.

### 4.13. Statistical Analysis

Statistical analyses were performed using SPSS 21.0 (SPSS, Inc., Chicago, IL, USA). The data in Table 1 and Table 2 and Figure 1 and Figure 2A were compared between different groups by ANOVA. Other data were compared between different groups by Student’s *t*-test. *p* < 0.05 was considered statistically significant.

## 5. Conclusions

To conclude, supplementation of SDF1 during IVM decreases oxidative stress, alters the expression of genes related to apoptosis and oocyte quality, and enhances the maturation and development potential of porcine oocytes. Our findings provide new insights into the mechanisms regulating oocyte IVM, and establish a new approach to improve the developmental potential of in vitro matured oocytes.

## Figures and Tables

**Table 1:** Effects of supplementation of SDF1 during IVM on maturation rate of pig oocytes.

Concentration (ng/mL)	No. of Repetition	No. of Cultured Oocytes	No. of Matured Oocytes (%, mean ± SEM)
0	6	650	458 (71.26 ± 0.91 ^a,c^)
1	5	487	357 (73.02 ± 1.63 ^a,b^)
20	6	645	492 (75.98 ± 0.70 ^b^)
100	5	448	308 (68.64 ± 1.47 ^c^)
200	3	204	139 (67.94 ± 1.59 ^c^)

Oocytes cultured with the IVM medium for 44 h and showing the first polar body in their perivitelline space were considered as mature oocytes. Values in the same column labeled with different superscripts differ at *p* < 0.05.

**Table 2:** Effects of supplementation of SDF1 during porcine oocyte IVM on subsequent development of SCNT embryos.

Concentration (ng/mL)	No. of Repetition	No. of Cultured SCNT Embryos	No. of Cleaved Embryos (%, mean ± SEM)	No. of Blastocysts (%, mean ± SEM)
0	4	184	137 (73.87 ± 1.15 ^a^)	34 (17.99 ± 2.81 ^a^)
1	4	172	131 (76.32 ± 2.78 ^a,b^)	30 (17.13 ± 0.97 ^a^)
20	4	159	132 (83.83 ± 2.88 ^b^)	47 (29.18 ± 3.20 ^b^)
100	4	170	141 (82.02 ± 3.21 ^b^)	38 (21.47 ± 2.82 ^a^)
200	3	132	102 (78.40 ± 2.62 ^a,b^)	27 (20.04 ± 1.25 ^a^)

Values in the same column labeled with different superscripts differ at *p* < 0.05.

**Table 3:** Effects of supplementation of SDF1 during porcine oocyte IVM on subsequent development of PA embryos.

Concentration (ng/mL)	No. of Repetition	No. of Cultured PA Embryos	No. of Cleaved Embryos (%, mean ± SEM)	No. of Blastocysts (%, mean ± SEM)
0	5	187	161 (86.51 ± 3.26)	95 (50.99 ± 1.38 ^a^)
20	5	196	183 (93.37 ± 1.02)	127 (64.77 ± 1.10 ^b^)

Values in the same column labeled with different superscripts differ at *p* < 0.05.

**Table 4:** Effects of supplementation of SDF1 during porcine oocyte IVM on subsequent development of IVF embryos.

Concentration (ng/mL)	No. of Repetition	No. of Cultured IVF Embryos	No. of Cleaved Embryos (%, mean ± SEM)	No. of Blastocysts (%, mean ± SEM)
0	4	154	112 (72.72 ± 0.78 ^a^)	28 (18.20 ± 1.16 ^a^)
20	4	134	112 (83.53 ± 2.67 ^b^)	42 (31.10 ± 1.41 ^b^)

Values in the same column labeled with different superscripts differ at *p* < 0.05.

**Table 5:** Summary of data generated by RNA-seq of porcine oocytes of the 0 ng/mL and 20 ng/mL SDF1 treatment groups.

Concentration (ng/mL)	Samples	Raw Reads (million)	Clean Reads (million)	Q30 (%)	Total Mapped Reads (million)	Total Unique Mapped Reads (million)	Percentage of Mapped Reads in Total Reads (%)	Percentage of Unique Mapped Reads in Mapped Reads (%)
	Control 1	53.79	51.94	93.81	35.74	34.90	68.81	67.20
0	Control 2	55.74	53.87	93.83	36.73	35.85	68.19	66.57
	Control 3	56.66	54.74	93.66	35.72	34.87	65.24	63.70
	Treatment 1	48.36	46.25	93.43	38.19	37.27	82.57	80.60
20	Treatment 2	53.40	51.58	93.60	35.99	35.16	69.79	68.17
	Treatment 3	54.65	52.48	93.36	37.88	36.97	72.19	70.44

## Data Availability

The corresponding author can provide access to the datasets upon request.

## Supplementary Materials

The following supporting information can be downloaded at: https://www.mdpi.com/article/10.3390/molecules27206830/s1, Table S1: The information of primers used for qPCR; Table S2: The DEGs between the SDF1-treated and control pig oocytes (see supplementary Excel document).
