# Supplementary material for: Supplementation of SDF1 during Pig Oocyte In Vitro Maturation Improves Subsequent Embryo Development
Source: Molecules. 2022 Oct 12;27(20):6830. doi: 10.3390/molecules27206830 (PMC9609306; doi:10.3390/molecules27206830)
Supplement: Supplementary file 1 [file molecules-27-06830-s001.zip › Table S1.pdf]

Table S1. The information of primers used for qPCR.

Genes	Genebank Accession No.	Primer Sequence
RN18S	NR_046261.1	F:5'- ACACGGACAGGATTGACAGATTGATAG-3'
(Internal control)		R:5'- TAACCAGACAAATCGCTCCACCAAC-3'
BAX	XM_013998624.2	F:5'- CCCTTTTGCTTCAGGGTTTCAT-3'
		R:5'- GCCGTCAGCAAACATTTCGG-3'
BCL2	XR_002346028.1	F:5'- CGTCCCAGCTCCACATCACC-3'
		R:5'- AGTGCCCCACCGAAGGAGAA-3'
GPX4	NM_214407.1	F:5'- ATTCTCAGCCAAGGACATCG-3'
		R:5'- TTTGACGTTGTAGCCAGCAG-3'
LOC102165589	XM_021079920.1	F:5'- GTGAAGGGCCAAAAGAGGAGTC-3'
		R:5'- CATGAGTGGCACGGATAATCTCG-3'
LYPD6	NC_010457.5	F:5'- ACCCTTCAACCACACCATATCC-3'
		R:5'- CTGTGACTTCCATTGTGTGCTG-3'
RAB43	NC_010455.5	F:5'- TACGACATCACCAAGAAGAGCTC-3'
		R:5'- GATGTCATAGTGCTCTGCCAGG-3'
SOD1	NM_001190422.1	F:5'- AAGGCCGTGTGTGTGCTGAA-3'
		R:5'- AGTGGCCACACCATCTTTGC-3'
SSMEM1	XM_003484018.4	F:5'- CCTACAAGCGGCAAAGCAAAGATG-3'
		R:5'- GAGTCAGTTACAGCGGTGAGTTGG-3'
TET1	NM_001315772.1	F:5'- TAATGGCAGCACAGTGGTCT-3'
		R:5'- GATGGCCCCGGATTTGATCT-3'
YY1	XM_021099699.1	F:5'- ATACCCGGCATCGACCTCTC-3'
		R:5'- AACATCTTTGTGCAGCCTTTGT-3'
